# InGaN Laser Diode with Spin-on-Glass Isolation Fabricated by Planarization and Etch-Back Process

**DOI:** 10.3390/mi17020142

**Published:** 2026-01-23

**Authors:** Katarzyna Piotrowska-Wolińska, Szymon Grzanka, Łucja Marona, Krzysztof Gibasiewicz, Anna Kafar, Piotr Perlin

**Affiliations:** Institute of High Pressure Physics, Polish Academy of Sciences, 01-142 Warsaw, Poland; kate_p7@mail.unipress.waw.pl (K.P.-W.); lucja@unipress.waw.pl (Ł.M.); kgibasiewicz@unipress.waw.pl (K.G.); ak@unipress.waw.pl (A.K.); piotr@unipress.waw.pl (P.P.)

**Keywords:** gallium nitride, SOG, lasers, planarization

## Abstract

We report on the fabrication and characterization of InGaN-based ridge-waveguide laser diodes employing spin-on-glass (SOG) as the insulation and planarization layer. In contrast to conventional silicon dioxide (SiO_2_) isolation deposited by PECVD, the SOG approach provides improved surface planarity, reduced processing complexity, and lower fabrication cost. The laser structures were grown on GaN substrates by MOCVD, with the active region consisting of In_0.11_Ga_0.89_N quantum wells. Following ridge formation and SOG deposition, an etch-back process was used to form the electrical contacts. We have demonstrated the formation of high-quality insulating surfaces with strong adhesion to the ridge sidewalls. When using a Ni protective layer, the fabricated devices exhibited favorable electrical and optical characteristics and achieved stable laser operation under both pulsed and continuous-wave conditions. These results indicate that the SOG-based insulation process represents a promising alternative for the scalable and cost-effective fabrication of InGaN laser diodes targeting advanced photonic applications.

## 1. Introduction

Group III-nitride semiconductors (InGaN, AlGaN, and GaN) are key materials for optoelectronic devices operating in the visible spectral range. Their direct bandgap, wide spectral tunability—from the near-infrared to the deep ultraviolet—and high thermal stability make them essential for applications such as solid-state lighting, disinfection, biomedical instrumentation, and emerging quantum technologies [1,2,3].

Blue InGaN-based laser diodes (LDs) represent the most technologically advanced class of nitride laser diodes, benefiting from the availability of bulk GaN substrates and the demonstration of high-power emission. They also exhibit exceptional robustness under extreme operating conditions [4,5], making them suitable for demanding applications, such as light sources for atomic clocks operating beyond Earth’s atmosphere. In such environments, device longevity is of critical importance.

**Figure 1:**
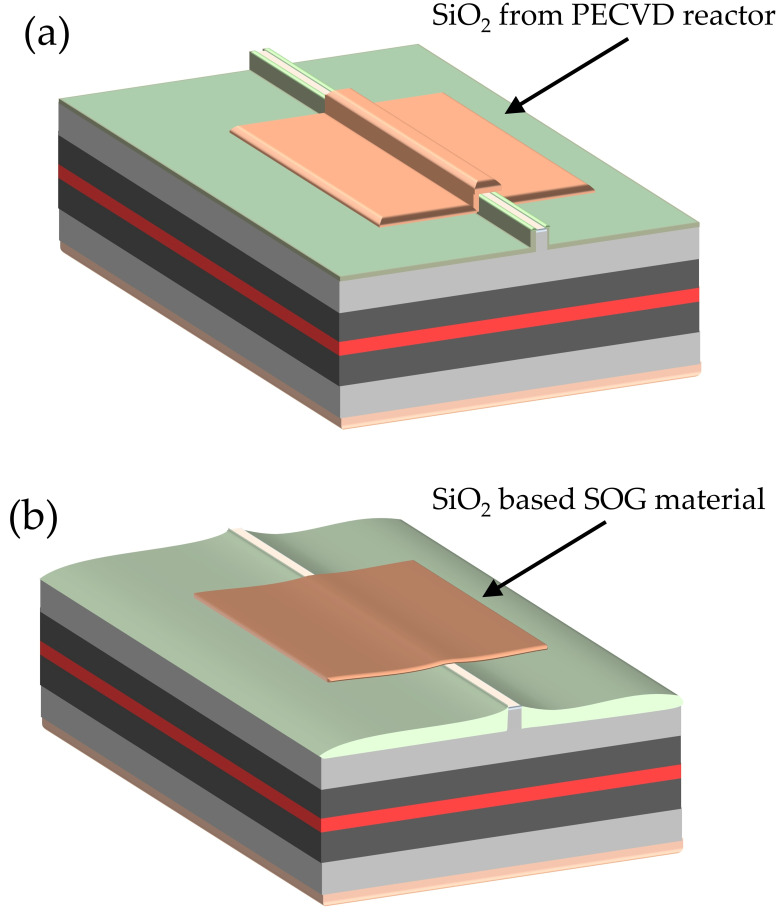
Schematic views of (**a**) oxide-isolated ridge-waveguide laser and (**b**) spin-on-glass (SOG) planarized ridge-waveguide laser diode.

In this work, we address one of the key fabrication steps that affects both the performance and reliability of InGaN laser diodes—electrical insulation. Conventionally, plasma-enhanced chemical vapor deposition (PECVD) silicon dioxide (SiO_2_) is used to electrically isolate ridge-waveguide LDs (Figure 1a). However, this approach suffers from several limitations. The deposited SiO_2_ typically exhibits relatively low material quality [6,7], characterized by surface roughness, non-uniformity, and a grain-like microstructure. These features can lead to electrical short circuits through the insulation layer, as well as adhesion and contamination issues, not only during deposition but also in subsequent processing steps. Moreover, the use of plasma during PECVD can damage the delicate top layers of the laser structure, particularly the sub-contact layer [8].

Finally, in the conventional PECVD-based processing scheme, the ridge top remains elevated relative to the surrounding wafer areas covered by SiO_2_. This non-planar surface morphology complicates p-side-down (flip-chip) mounting of laser chips, while the uneven topography adversely affects heat dissipation and mechanical stability [9].

Spin-on-glass (SOG) materials provide a compelling alternative to PECVD-deposited SiO_2_ due to their excellent gap-filling capability and superior surface smoothness (Figure 1b). Upon thermal curing, SOG forms a robust, low-k dielectric that is fully compatible with standard lithographic and etching processes. Unlike PECVD oxides, SOG processing avoids exposure to high-energy plasma, thereby minimizing damage to sensitive layers within the laser structure.

Methylsilsesquioxane (MSQ), a widely used SOG material, is well known for its effectiveness as both an insulating and planarization layer [10,11]. The MSQ layer efficiently electrically isolates the semiconductor material—including mesa sidewalls—from metal contacts and contact pads, which is essential for controlled current injection. This isolation prevents undesired current leakage outside the ridge-waveguide region. Moreover, planarization using an MSQ layer does not reduce the mesa height; instead, it compensates for surface topography non-uniformities by filling the regions surrounding the mesa, resulting in a more uniform and planar surface profile [12].

In this study, we investigated laser structures grown on GaN substrates by metal–organic chemical vapor deposition (MOCVD), with the active region consisting of In_0.11_Ga_0.89_N quantum wells. The use of MSQ insulation enabled the formation of high-quality dielectric layers exhibiting smooth surfaces and strong adhesion to ridge sidewalls. Electrical and optical characterization demonstrated stable laser operation under both pulsed and continuous-wave conditions. These results confirm that the SOG-based isolation process represents a promising, scalable, and cost-effective approach for the fabrication of InGaN laser diodes targeting advanced photonic applications.

## 2. Materials and Methods

For the SOG insulation layer, we selected Accuglass 512B (Honeywell Electronic Materials, 6760 West Chicago Street, Chandler, AZ, USA), a commercially available methylsiloxane-based spin-on-glass material that is chemically similar to methylsilsesquioxane (MSQ) and can therefore be regarded as a functional analogue. The material used in this work belongs to the MSQ family and is composed of –CH_3_-substituted Si–O units that link together to form a highly cross-linked silsesquioxane network, providing the characteristic chemical stability and excellent planarization properties of MSQ-based dielectrics.

Recent reviews [10,11,13] highlight MSQ’s strong potential as a next-generation low-k dielectric. Owing to its low dielectric constant, high planarization capability, and simple, scalable deposition via spin coating, MSQ is widely employed in advanced micro- and optoelectronic technologies. Fabrication routes described in patents confirm the industrial scalability of MSQ-based SOG processes; however, devices reported in the literature have predominantly been fabricated on silicon substrates, with methylsiloxane-based SOGs used primarily as planarization materials [14,15].

Accuglass 512B was selected because its organically modified methylsilsesquioxane (MSQ)-based chemistry provides a unique combination of properties, including high planarization efficiency—manifested by significant step-height reduction and effective gap filling—good mechanical strength, excellent etch-back controllability, and thermal compatibility with InGaN-based laser processing. Such a combination cannot be achieved with inorganic SOG formulations. The organic modification of Accuglass 512B enables step-height reduction of up to 700 nm per layer (equal to the layer thickness) at moderate spin-coating speeds, which meets the minimum requirement for InGaN edge-emitting laser fabrication. Thinner coatings would require multiple deposition and curing cycles, increasing the risk of stress accumulation and defect formation. Under SF_6_-based ICP etching conditions, MSQ-based materials exhibit an etch rate of approximately 200 nm min^−1^, allowing controlled and reproducible exposure of the ridge top. In contrast, silicate-based SOGs, which are chemically closer to SiO_2_, etch roughly an order of magnitude more slowly under comparable conditions. Although Accuglass 512B has a nominal curing temperature of 425 °C, it remains thermally stable up to approximately 500–550 °C, which is sufficient for Ni/Au contact formation. By comparison, silicate-based SOGs require densification temperatures of about 600–700 °C, significantly increasing the risk of metal–GaN interfacial reactions and degradation of the p-GaN subcontact layer.

The integration of MSQ-type SOG materials into III-nitride laser diode processing is not straightforward. Despite their established role in microelectronics, to the best of our knowledge no studies have reported the use of MSQ-derived SOG materials in wide-bandgap laser diodes. This gap is likely attributable to several intrinsic vulnerabilities of MSQ, including limited thermal stability [10], moisture uptake [16], and potential chemical and mechanical interactions with metal contacts, particularly when compared to conventional PECVD-deposited SiO_2_ or silicate-based compounds.

Moisture uptake in MSQ originates from its inherent nanoscale porosity, the presence of residual –OH groups, and incomplete cross-linking [16]. Thermal stability is constrained by the decomposition of –CH_3_ substituents and by the relatively low-density Si–O–Si network, which may undergo shrinkage or cracking at temperatures exceeding approximately 450 °C. Furthermore, metal–MSQ interfaces may be susceptible to adsorption phenomena, chemical reactions, and mechanically induced stress arising from thermal expansion mismatch, potentially leading to increased leakage currents or interfacial delamination.

In contrast, silicate-type SOG materials, while still capable of providing planarization, typically exhibit slower etch rates, reduced mechanical compliance, and somewhat weaker adhesion due to their more inorganic, Si–O–Si-dominated network. Such structures tolerate mechanical stress less effectively but generally offer slightly improved thermal resistance compared to pure MSQ-class materials.

Overall, pure MSQ formulations—including Accuglass 512B—offer a favorable combination of high planarization efficiency, fast and controllable etch-back behavior, and good mechanical compliance, making them particularly attractive for applications requiring precise material removal and effective surface leveling. Silicate-type SOGs, by contrast, trade etchability and mechanical flexibility for enhanced thermal robustness. Accordingly, this work focuses on establishing a reliable processing window for Accuglass 512B in III-nitride ridge-waveguide laser diodes, with particular emphasis on controlling SOG densification during sequential annealing, preserving layer integrity during SF_6_ plasma etch-back, and evaluating the resulting impact on device performance. For clarity, in the following sections the abbreviation “SOG” refers exclusively to the commercial spin-on-glass material Accuglass 512B.

The primary challenge of this study arises from the fact that, during various laser fabrication steps, the SOG material is exposed to elevated temperatures, aggressive plasma processes, and metal-contact annealing (e.g., Ni/Au). Such conditions can induce structural changes in the film, including densification, degradation of methyl groups, or metal diffusion. Nevertheless, through careful optimization of the processing sequence, no significant moisture uptake or material instability was observed. This successful outcome is attributed to the three-stage thermal treatment implemented in our process, which is described in detail below.

After extensive testing, the following process recipe was selected. Honeywell Accuglass 512B was spin-coated at 2000 rpm under ambient air conditions. The as-deposited films were subsequently soft-baked on a hot plate using a three-step procedure: 80 °C for 1 min, followed by 150 °C for 1 min, and finally 250 °C for 10 min. This gradual heating strategy is critical, as rapid temperature increases can generate thermal stress in the still-polymeric, solvent-containing film, leading to cracking or delamination. The samples were then fully cured in a rapid thermal processing (RTP) system at 425 °C for 60 min under a nitrogen atmosphere. Under these spin-coating and soft-baking conditions, a target film thickness of 780 nm was obtained, as confirmed by scanning electron microscopy.

RTP curing in N_2_ promotes siloxane condensation, solvent removal, and network formation and represents a standard processing step for MSQ-based materials. However, subsequent high-temperature annealing at 500 °C in ambient air—performed during metal contact formation—induces additional cross-linking and densification of the Si–O–Si network. This annealing step, which is non-standard for Accuglass materials, occurs at temperatures above ~450 °C, where partial decomposition of organic groups and a reduction in free volume are known to take place, yielding a structure more closely resembling dense SiO_2_. The 500 °C air anneal promotes oxidation and removal of residual organic groups [10,16], further enhances network cross-linking, and increases film density. As a result, the final dielectric layer exhibits significantly improved thermal robustness and a markedly reduced susceptibility to humidity compared to conventionally reported MSQ films.

The successful implementation of SOG in InGaN laser diode processing also required modifications to the standard fabrication scheme. In conventional InGaN ridge-waveguide laser processing, ridge isolation is typically formed using a self-aligned lift-off procedure, in which the same photoresist pattern defines both the ridge geometry and the dielectric isolation. Following contact layer deposition across the wafer, stripe photoresist patterns are defined, the ridges are etched, the dielectric layer is deposited, and a lift-off step removes the photoresist along with the dielectric from the ridge top, exposing the sub-contact layer while leaving the ridge sidewalls insulated. When using SOG, however, this lift-off approach is not applicable, as SOG films are prone to cracking during resist removal. Instead, when SOG is spin-coated onto a wafer with pre-etched ridges, the resulting film thickness on the ridge tops is significantly lower than in the regions between the ridges. This behavior contrasts sharply with that of PECVD-deposited oxides, which typically exhibit uniform thickness across the entire wafer surface.

Consequently, the SOG layer can be selectively thinned using an SF_6_ plasma etch-back process, which removes a uniform thickness of material across the wafer, thereby exposing the mesa top while preserving electrical insulation on the ridge sidewalls and in the regions between adjacent ridges. The etch-back process reduces the overall surface height to uncover the mesa top, enabling subsequent metal contact deposition [11,14]. This technique is commonly employed to expose underlying structures, such as ridges or contact regions.

**Figure 2:**
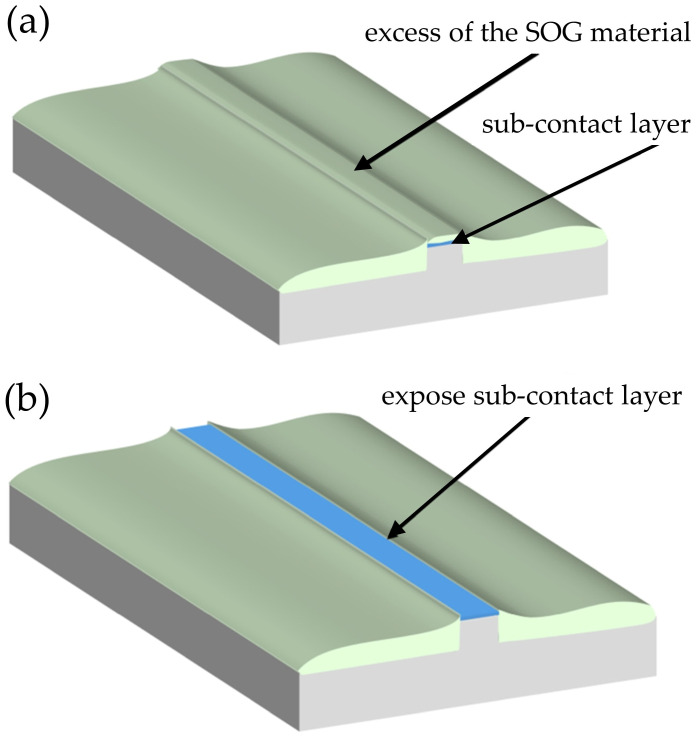
Schematic of the etch-back process (**a**) before the excess SOG material was removed, (**b**) after exposure to the SF_6_ plasma, the top of the ridge is exposed.

Following SF_6_ plasma exposure, the ridge top becomes uncovered and directly accessible for further processing. As illustrated schematically in Figure 2, removal of the excess SOG material is a critical step for continued laser diode fabrication. However, direct exposure of the ridge top to SF_6_ plasma may adversely affect the surface and its electrical properties (Figure 2b). In particular, SF_6_ etching can lead to fluorine incorporation into p-type GaN:Mg, resulting in the formation of electrically insulating surface layers on top of the ridge [17].

A second major challenge arises from the inability to employ the conventional self-aligned process in which the metal contact is deposited directly on top of the ridge and serves to protect the p-GaN sub-contact layer during etching. In the present approach, this strategy is not applicable because the optimized SOG fabrication procedure requires a final annealing step at 425 °C. Since oxygen availability is essential for proper ohmic contact formation [18,19], additional annealing under an SOG cap—which restricts oxygen diffusion—leads to degradation of the metal contacts.

To address this issue, we introduced a removable protective layer deposited on top of the wafer prior to ridge etching. This approach enables precise coverage of only the ridge top and allows the SOG layer to be etched back without exposing or damaging the underlying sub-contact layer.

To identify the most suitable material for this protective function, we investigated a set of metal layers, including Ni, Al, Cr, Pt, and a Ni/Au bilayer. The performance of each candidate was evaluated by measuring the electrical properties of the resulting metal contacts using the transfer length method (TLM) after removal of the protective layer. A key requirement was that the presence and subsequent removal of the protective material must not degrade the final device parameters. In addition, the ease and cleanliness of protective-layer removal were assessed. The ideal metal mask for the p-GaN sub-contact layer should exhibit a low etch rate in SF_6_ plasma, be readily removable after oxygen-limited SOG annealing, and show no detrimental chemical interaction with GaN.

An additional consideration arises from the fact that, during high-temperature SOG curing, the metal masks are located beneath the SOG layer. In this configuration, the SOG acts as an oxygen diffusion barrier, causing the metals to anneal under oxygen-restricted conditions. In such a semi-sealed environment, the chemical behavior of the metals—including oxidation pathways—differs significantly from that in open air. While previous studies have examined NiO_X_-based contact formation and the role of p-GaN surface oxides, the combined effects of metal-mask oxidation and SOG-limited oxygen transport have not been systematically investigated. This gap is particularly important because metals such as Al and Cr undergo volumetric oxidation, whereas Ni forms a self-limiting oxide. These distinct oxidation behaviors may fundamentally influence the p-GaN surface chemistry and the subsequent formation of NiO_X_ ohmic contacts. Accordingly, it is essential to determine how temporary metal masks (Ni, Al, Cr, and Ni/Au) behave during SOG-assisted annealing and how their behavior affects p-GaN surface properties and final contact performance.

**Figure 3:**
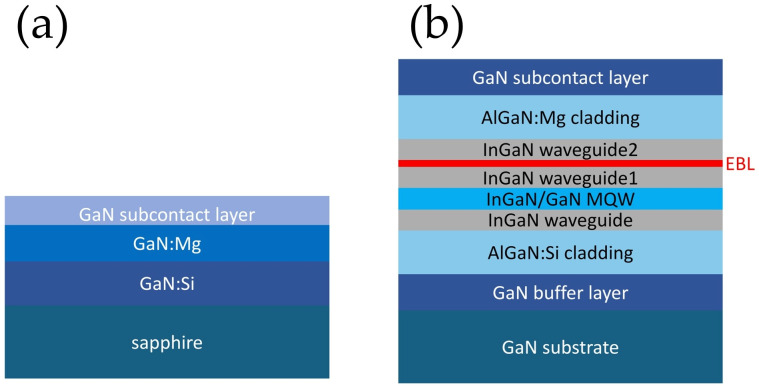
Epitaxial structures used in the experiment: (**a**) test structure for TLM, (**b**) full laser structure.

We first prepared test epitaxial structures (simple p-GaN test layers), in an Aixtron CCS 3x2 metalorganic vapor phase epitaxy (MOVPE) reactor (AIXTRON SE, Dornkaulstr. 2, Herzogenrath, Germany). The test structures were grown on sapphire substrates and consisted of a 1 µm GaN:Si layer, followed by a 0.5 µm GaN:Mg layer and a highly doped p^+^-GaN subcontact layer (Figure 3a). The Mg concentration was in the range of 2–5 × 10^18^ cm^−3^ in the main layer and about 1 × 10^20^ cm^−3^ in the subcontact layer.

**Figure 4:**
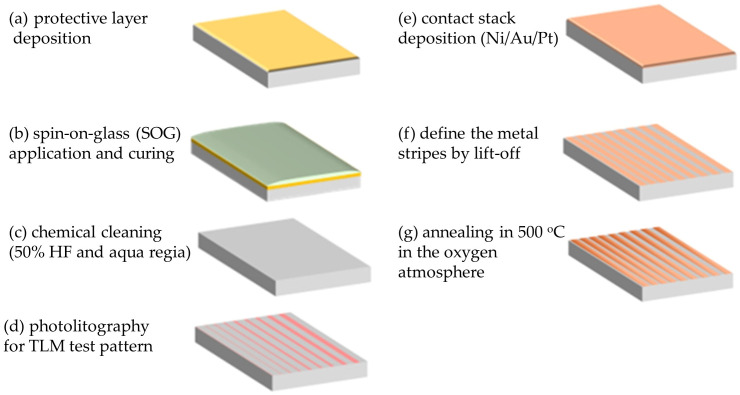
Main steps in the fabrication of TLM test devices with a protective metal layer.

A simplified processing sequence, excluding mesa etching, was then implemented, as schematically illustrated in Figure 4. Each selected metal protective layer was first deposited on a p-GaN wafer (Figure 4a), followed by spin coating and annealing of the SOG layer. A critical step in this test was the final annealing at 425 °C performed beneath the SOG layer in order to simulate oxygen-limited conditions (Figure 4b). Subsequently, the SOG layer was completely removed by wet etching in hydrofluoric acid for 5 min, after which the protective metal layer was stripped using aqua regia (Figure 4c). Photolithography was then used to define a transfer length method (TLM) test pattern (Figure 4d). A standard metal contact stack (Ni/Au/Pt) was deposited (Figure 4e), followed by lift-off (Figure 4f). The contacts were annealed at 500 °C (Figure 4g), and TLM characterization was performed [20]. The spacing between adjacent metal pads ranged from 5 µm to 45 µm in increments of 5 µm. Based on this experimental procedure and the electrical parameters obtained (presented in the Results section), Ni was identified as the most suitable protective metal for SOG-based InGaN laser diode processing.

Subsequently, laser diode fabrication was carried out. The epitaxial structure was grown in the same MOVPE reactor; however, in this case, a commercial hydride vapor phase epitaxy (HVPE) GaN substrate was used. The laser structure (Figure 3b) was designed as a separate-confinement heterostructure with graded-composition cladding layers [21]. Growth began with a 2 µm Si-doped GaN buffer layer, followed by a 1190 nm Al_0.075_Ga_0.925_N:Si bottom cladding layer and a 150 nm In_0.045_Ga_0.955_N:Si bottom waveguide. The active region consisted of two 3.5 nm In_0.11_Ga_0.89_N quantum wells separated by 6 nm GaN barriers. The upper part of the structure comprised a 50 nm In_0.045_Ga_0.955_N waveguide, a 20 nm AlGaN:Mg electron-blocking layer (EBL), a 100 nm In_0.045_Ga_0.955_N:Mg top waveguide, a 650 nm Al_0.05_Ga_0.95_N:Mg cladding layer, and GaN:Mg contact and sub-contact layers with thicknesses of 200 nm and 10 nm, respectively. The doping level of the sub-contact layer was identical to that used in the TLM test structures.

**Figure 5:**
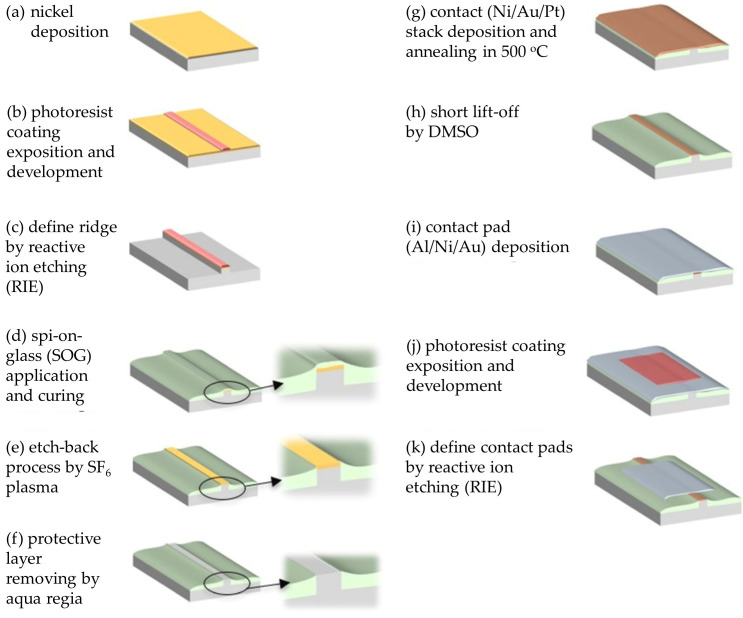
Main steps of laser diode fabrication by SOG-based planarization and etch-back process.

The laser diode processing sequence is summarized in Figure 5. First, a nickel layer was deposited on the laser wafer (Figure 5a), patterned by photolithography to define ridge stripes (Figure 5b), and subsequently etched (Figure 5c). Ridge waveguides were formed using Cl_2_/Ar inductively coupled plasma reactive-ion etching (ICP-RIE). After photoresist removal, the structure was planarized using Accuglass 512B (Figure 5d), and the SOG layer was etched back to expose the ridge tops (Figure 5e). The controlled etch-back process was performed in an ICP reactor using SF_6_ plasma at an ICP power of 350 W and an RF power of 40 W for 3 min at 60 °C. Following etching, the Ni protective mask was removed using aqua regia (Figure 5f). Final p-side metallization was then deposited and annealed to reduce contact resistance (Figure 5g), after which laser stripes were defined by a lift-off process (Figure 5h). The top metal contact stack Al/Ni/Au was subsequently deposited (Figure 5i). A second photolithography step was used to define the contact pads (Figure 5j), which were formed by argon dry etching in an ICP-RIE system (Figure 5k), followed by resist removal. On the backside, the wafer was thinned to approximately 100 µm and metallized with a Ti/Al/Au contact stack. After completion of processing, the wafer was cleaved and diced, and individual laser chips were mounted in TO-56 packages. The fabricated devices had a ridge width of 3 µm and a resonator length of 750 µm.

## 3. Results

### 3.1. Electrical Measurements and Surface Characterization of TLM Structures p-GaN

**Figure 6:**
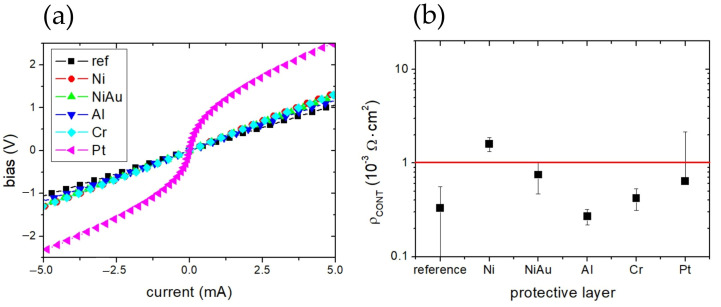
(**a**) I–V characteristics for 5 μm distance between contact pad and (**b**) contact resistance of TLM devices processed with different protective layers (the red line indicates the acceptable contact resistance value-for better readability only).

The transfer length method (TLM) patterns fabricated on the p-GaN test structures (Figure 3a) were electrically characterized. Representative current–voltage (I–V) characteristics measured for a contact spacing of 5 µm are shown in Figure 6a. The measurements were performed after removal of the temporary metal protective layers. For nearly all samples, the I–V characteristics exhibited good linearity (Figure 6a) and low specific contact resistivity, on the order of ~1 × 10^−3^ Ω·cm^2^ (Figure 6b). The only exception was the Pt-protected sample, which showed a distinctly non-linear I–V characteristic. Notably, samples previously protected with Al or Cr exhibited significantly lower contact resistivity compared to the reference sample, whereas Ni-protected samples showed a slight degradation in TLM performance. It is important to emphasize that all TLM structures employed the same final Ni/Au/Pt contact stack. Consequently, the observed differences in electrical behavior do not arise from the final metallization itself, but rather from variations in the condition of the p-GaN surface following removal of the different temporary metal protective layers.

**Figure 7:**
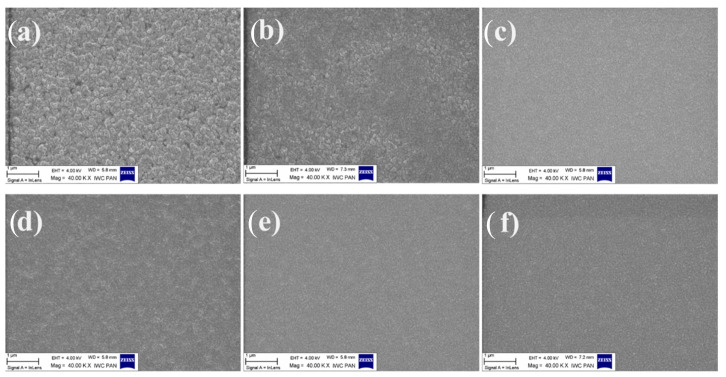
SEM analysis of the Ni/Au/Pt contact stack after SOG mask removal; (**a**) Ni, (**b**) Ni/Au, (**c**) Pt, (**d**) Al, (**e**) Cr, and (**f**) without any deposited metal.

To further investigate these effects, scanning electron microscopy (SEM) measurements were performed (Figure 7). The surface morphology of the final Ni/Au/Pt contact stack was compared for samples that underwent identical lithography and lift-off procedures and differed only in the type of the initial metal protective layer. Aqua regia successfully removed Al-, Ni-, and Pt-based protective layers. In contrast, removal of Cr required an additional etching step to achieve a uniform p-GaN surface. The reference sample, in which the Ni/Au/Pt contact was formed without any protective layer, exhibited the most homogeneous SEM appearance. Contacts fabricated after removal of Ni-, Ni/Au-, and Al-based protective layers showed a characteristic grainy and non-uniform surface texture. In comparison, contacts formed after removal of Cr- and Pt-based protective layers displayed a more uniform SEM contrast. Despite its visually smooth morphology, the Pt-based contact exhibited weaker adhesion to the p-GaN surface.

Nickel was selected as the protective layer for our fabrication process, due to its predictable chemical behavior during removal after the etch-back process. During the SF_6_-based etch, reactive fluorine radicals generated in the plasma [22] readily interact with exposed metal surfaces. Compared to other used metals such as Cr, Al, or Pt, the nickel fluorides forms, such as NiF_2_ can be effectively removed by wet etching, leaving a uniform p-GaN surface [23,24,25].

The selection of Ni as the protective layer was based on several key considerations: compatibility with the existing fabrication process, ease of removal using aqua regia, favorable current–voltage characteristics, and acceptable contact resistance. Alternative metals were excluded for the following reasons: non-ohmic current–voltage behavior and poor adhesion in the case of Pt, incompatibility with the established process flow due to the need for additional removal steps for Cr, and surface inhomogeneities potentially arising from the formation of insulating fluorides in the case of Al.

A temporary thin Ni layer deposited in the island (Volmer–Weber) growth mode can exhibit nanometer-scale discontinuities. These discontinuities may partially expose the p-GaN surface to fluorine species during plasma processing, leading to the formation of electrically resistive GaF_X_ and Mg–F compounds and, consequently, an increase in the operating voltage. Fluorine-related degradation can be mitigated primarily by improving protection of the p-GaN subcontact layer, for example, by increasing the mask thickness (to approximately 50 nm), thereby enhancing mask continuity, and by minimizing plasma exposure.

### 3.2. SOG Planarization and Isolation Quality

**Figure 8:**
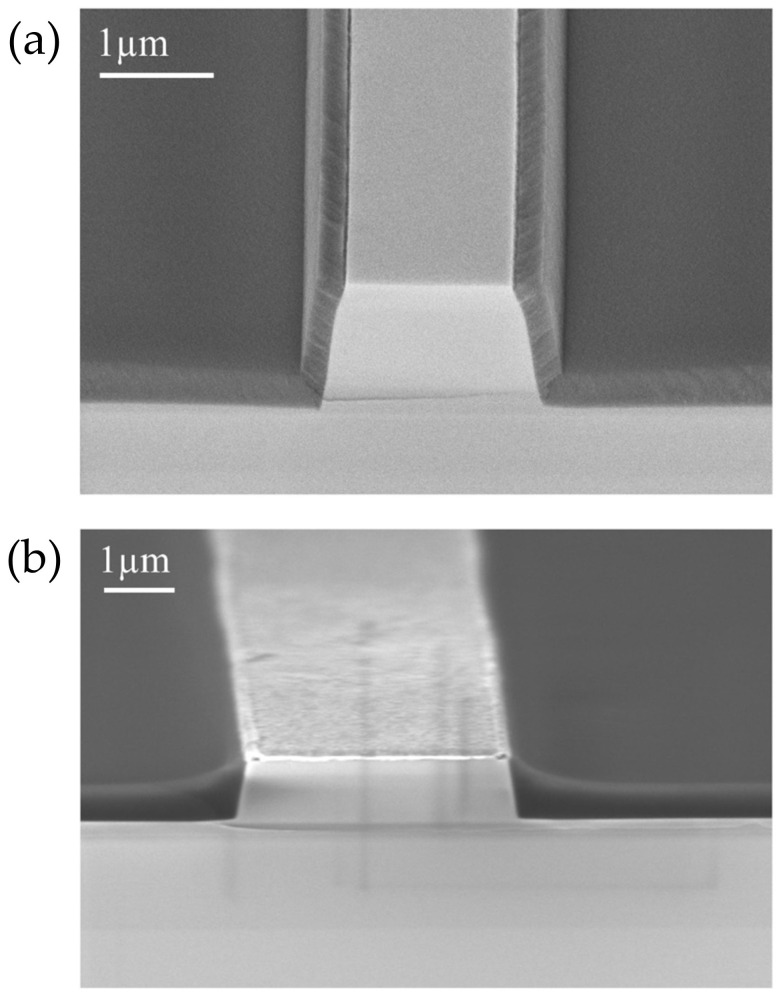
Cross-sectional SEM images of (**a**) LD with SOG-based planarization and (**b**) LD without planarization (SiO_2_ isolation by PECVD).

Figure 8 compares mesas isolated using conventional PECVD-deposited SiO_2_ and SOG-based insulation. The mesa shown in Figure 8a was fabricated with a slightly narrower ridge width (2 µm) than that of the SOG-insulated structure (3 µm); however, in both cases the etch depth was 800 nm. Cross-sectional SEM images clearly demonstrate a substantial improvement in surface planarity for the SOG-based process compared with PECVD SiO_2_ isolation. The step height reduction (SHR) achieved using SOG planarization was approximately 56% [12]. As shown in Figure 8b, the SOG coating provided effective planarization of the ridge region: although the surface is not perfectly flat, the overall topography is significantly more uniform, and the ridge top was uniformly covered and subsequently exposed during the etch-back step. This reduction in step height facilitates more uniform metal deposition and reduces mechanical stress in the final device stack. The SOG layer coated the wafer uniformly and exhibited good adhesion to the ridge surfaces. No cracks or delamination were observed, indicating the mechanical integrity and reliability of the SOG-based insulation process.

### 3.3. Optical Characterization of Laser Diode Isolated by SOG

**Figure 9:**
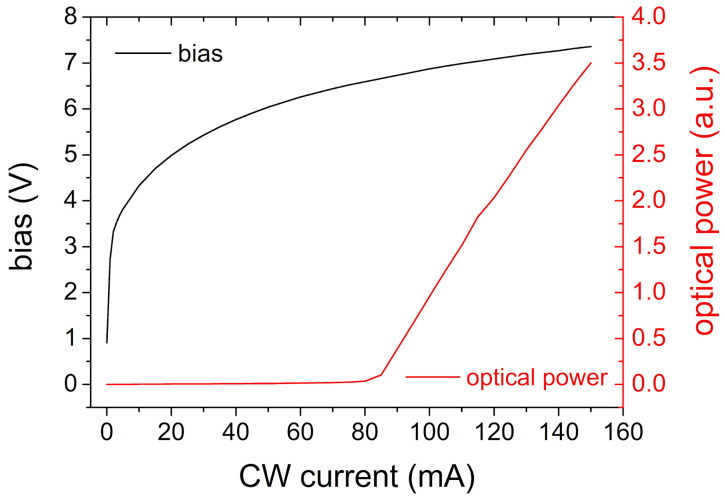
Current–voltage (I-V) characteristics of SOG-planarized InGaN laser diodes under CW operation.

The fabricated SOG-isolated laser diodes were characterized in both continuous wave (CW) and pulsed operation under temperature stabilization to 20 °C by a thermoelectric cooler. The devices lased at 435 nm with threshold currents around 80 mA (≈5.7 kA/cm^2^) and voltages of ~6.5 V (Figure 9). The observed voltage is higher than in conventionally processed LDs, and the origin of this increase is under investigation. We observed stable emission under both pulsed and CW operation. To our knowledge this is the first demonstration of the successful implementation of a SOG isolation in InGaN laser diodes.

## 4. Discussion

The results presented in this work demonstrate that methylsilsesquioxane (MSQ)-based spin-on-glass (SOG) is a viable alternative to PECVD SiO_2_ for electrical isolation of InGaN ridge-waveguide laser diodes. The SOG planarization process reduced the surface step height by approximately 56%, yielding a significantly more uniform topography compared to conventional oxide isolation. This improved surface morphology facilitates high-quality metal deposition, reduces mechanical stress in the p-contact stack, and enhances compatibility with advanced assembly techniques, such as p-side-down mounting. Furthermore, stable continuous-wave lasing confirms that the SOG fabrication process is fully compatible with InGaN laser diode processing.

A key aspect of SOG integration is the need to expose the ridge top through SF_6_-based etch-back process. Fluorine incorporation into p-GaN:Mg is known to generate thin, highly resistive surface layers, which may increase the effective series resistance of the device [17]. This mechanism is consistent with the elevated operating voltage (~6.5 V) observed in the SOG-processed lasers compared with typical values for similarly designed InGaN LDs. The increased voltage does not prevent lasing but indicates that further optimization of the etch-back and post-etch surface preparation will be required to achieve the lowest possible electrical resistance in future devices. Although in our experiment Ni nominally protects the ridge top during the SF_6_ etch-back, it may not be continuous. Thin Ni films deposited on GaN are known to grow in a Volmer–Weber (island) mode [26,27,28], exhibiting nanoscale discontinuities and pinholes even at nominal thicknesses around 25 nm. Under such conditions the Ni layer behaves as a partially perforated mask rather than a continuous barrier. As a result, local etch-rate nonuniformities, mask thinning, or ion-induced sputtering during SF_6_ exposure can transiently open sub-micron pathways through which reactive fluorine species reach the underlying p-GaN surface. Even momentary fluorine ingress is sufficient to form GaF_X_ or Mg-F species, both of which are electrically insulating and difficult to remove in subsequent cleaning steps. These fluorinated surface layers increase the effective series resistance and are consistent with the elevated operating voltage observed in SOG-processed laser diodes. However, increasing the Ni thickness to eliminate pinholes introduces a competing limitation. Thick Ni layers (>50–70 nm) significantly improve the film continuity and robustness against sputtering, but they complicate wet removal and increase the risk of residue formation, particularly when the mask is capped by an oxidized or roughened surface after high-temperature curing beneath the SOG layer. This creates a practical trade-off between ensuring sufficient barrier integrity during SF_6_ etching and maintaining reliable, damage-free mask removal prior to ohmic-contact deposition. A balanced solution—supported by both literature and our processing experience—is to employ intermediate Ni thicknesses (≈40–60 nm) or a Ni/Au bilayer, in which a thin Au cap increases the surface continuity and suppresses sputtering while keeping the overall mask readily removable in aqua regia. Such intermediate-thickness masks minimize the probability of fluorine penetration during etch-back without introducing complications associated with thick-metal lift-off or etch residue.

In parallel with the planarization studies, we investigated the behavior of temporary metal protection layers during SOG curing and their influence on the surface condition of the p-GaN sub-contact layer. Although metal masks are commonly used for mesa protection, their response under oxygen-restricted annealing—induced here by the SOG overlayer—has not been systematically examined. Transfer-length-method (TLM) measurements revealed unexpectedly low contact resistance for Al- and Cr-protected samples, whereas Ni-protected samples exhibited only a slight increase in resistance compared to the reference. Two competing explanations may account for these observations. The first suggests that Al and Cr undergo volumetric oxidation during SOG-capped annealing, promoting oxidation of the underlying p-GaN. Subsequent HF cleaning then produces an ultrathin, chemically uniform fluoride layer that homogenizes the interface, artificially lowering the TLM-extracted contact resistivity. In this scenario, the low TLM values do not indicate improved ohmic behavior but rather reflect a smoother chemical landscape that masks the true interface quality [20]. This interpretation aligns with previous reports showing that Cr-containing contacts typically exhibit degraded ohmic performance due to interfacial oxidation [29]. The second possibility is that Al and Cr act as chemical getters, removing surface contaminants or native oxides and thereby producing a genuinely cleaner and more uniform interface [30]. Importantly, regardless of the underlying mechanism, Ni remains the most technologically reliable protective layer. Ni forms only a thin, self-limiting oxide during SOG curing, can be cleanly removed by wet etching, does not induce long-range chemical reconstruction of the p-GaN surface, and is fully compatible with standard Ni/Au/Pt ohmic-contact schemes.

The optical results further support the compatibility of the SOG-based process with high- performance laser diode fabrication. The devices fabricated here exhibit stable mode emission from a 3 µm ridge, confirming that neither the SOG planarization nor the modified surface treatment introduce detrimental waveguiding anomalies, filamentation, or lateral current spreading. To our knowledge, this constitutes the first demonstration of an InGaN laser diode using MSQ-based SOG as the primary isolation and planarization material.

Several limitations of the present study should be acknowledged. First, no chemical surface analysis was performed after SOG curing and protective-metal removal, which prevents unambiguous identification of the interfacial species responsible for the TLM behavior. Second, only Ni was used in full laser-diode processing; while Al- and Cr-protected devices showed promising TLM trends, their true suitability can only be evaluated by fabricating complete laser structures. This is because, unlike the electrically isolated TLM structures, the laser diode provides a direct measure of the true contact performance under vertical current injection. Third, the higher operating voltage indicates that further optimization of the SF_6_ etch-back and post-etch cleaning is needed. Finally, long-term reliability tests were not performed, although they are essential for evaluating the stability of MSQ-based dielectric layers under laser-diode operating conditions.

Despite these limitations, the present results underscore the technological potential of SOG-based isolation for nitride photonics. This approach provides improved surface planarity, avoids plasma-induced damage associated with PECVD, reduces process complexity, and is inherently scalable for large-area fabrication. When combined with a suitable temporary metal mask—identified here as Ni—the SOG-based planarization process offers a robust and cost-effective alternative for InGaN laser diode manufacturing. Future work will focus on optimizing the etch-back process, exploring alternative mask materials in complete laser diode fabrication, and conducting lifetime testing to evaluate long-term device stability.

## 5. Conclusions

In this work we demonstrate InGaN laser diodes fabricated using SOG ridge isolation, which enables partial planarization of the mesa topography and facilitates high-quality p-contact formation. The motivation for implementing SOG was to obtain smoother ridge profiles, improved surface morphology and reduced step height, all of which are essential for reproductible and low-resistance ohmic contacts.

Under these conditions, however, the choice of temporary metal mask becomes a critical factor because SOG creates a locally oxygen-restricted environment that can amplify mask-dependent surface reactions. As a result, Al and Cr probably promote p-GaN oxidation and form uniform fluoride layers that artificially reduce the TLM contact resistance, making TLM analysis unreliable. In contrast, Ni does not modify p-GaN chemistry and therefore allows proper NiO_X_ ohmic contact formation.

Consequently, only Ni-protected samples produced fully functional laser diodes with satisfaction electrical and optical characteristics. Taken together, these results demonstrate that Ni is, at present, the only temporary mask compatible with SOG-assisted InGaN laser fabrication. Further optimization is needed to lower the operating voltage, which will be the focus on future work. Nevertheless, the presented devices clearly show the strong potential of spin-on-glass for InGaN laser diode fabrication.

## Data Availability

The original contributions presented in this study are included in the article. Further inquiries can be directed to the corresponding author.
